# Work for the Wounded of the Allied Armies

**Published:** 1914-11-07

**Authors:** 


### Work for the Wounded of the Allied Armies.

**(From our Special Correspondents.)**

### MORE CONVOYS AT CAMBRIDGE.

ABOUT 174 wounded soldiers from the seat of war were brought to Cambridge in a Great Western Red Cross train on October 26. It is but a few weeks since they left the town for the Front. About 156 of the soldiers were conveyed in motor ambulances and cars to the 1st Eastern General Hospital. The remainder, consisting of officers, were conveyed to the Research Hospital, which has been placed at the disposal of the War Office for the reception of wounded officers. Mr. W. H. Bowen, M.D., M.S.(London), F.R.C.S.(England), surgeon, who is in charge of the nose, throat, and ear department at Addenbrooke's Hospital, is attending these wounded officers.

The Voluntary Aid Detachments in attendance at the station were as follows:—The Cambs. No. 16 (Women) from Linton, Cambs., and the surrounding district, the Cambs. No. 30 (Women) from Sawston and the villages around, the Cambs. No. 3 (Men) from Soham, and the Cambs. No. 13 (Men) from Cambridge. The Linton detachment included Commandant Mrs. F. Smith, Deputy Commandant Miss Smith, Lady Superintendent Nurse Church, Quartermaster Miss L. Stainton. The Sawston detachment was represented by Commandant Miss M. O. Briggs (Babraham), Mrs. Philip Arnold (Whittlesford) Quartermaster, and Nurse Hulme Superintendent. The Soham detachment comprised sixteen men, under Acting Commandant Dr. Cory. The Cambridge detachment was over sixty in number, under the command of Mr. W. H. Jennings. Dr. Johnson, medical attendant to the detachment, was also present.

Later in the day on Monday the officer in command at the 1st Eastern General Hospital received information that more wounded soldiers from the Front were being dispatched to Cambridge. Just before 2 A.M. on Tuesday the train "travelling hospital" steamed into the station, bringing about 170.

Scarcely had these 170 wounded soldiers been removed from the station to the hospital when a London and South Western ambulance train steamed into the station about 4.40, bringing another 100. These again included mostly men of the 6th Division, which was but a short time since encamped on Midsummer Common. The men were conscious of the fact that they would soon have the privilege of renewing the friendships which they made during their former stay in Cambridge. Their destination was the 1st Eastern General Hospital. Great assistance was rendered at the station by members of the Cambs. No. 13 (Men) Voluntary Aid Detachment, under Commandant W. H. Jennings.

### HUDDERSFIELD: ROYAL INFIRMARY.

One hundred wounded soldiers arrived in Huddersfield from Southampton on Friday last week. Of these, fifty-six are being treated in Number 4 Ward at this hospital. In addition nine have been sent to Miss Johnson's Private Nursing Home in Trinity Street, and five to Mrs. Taylor's Surgical Home in Bradley Lane, all under the care of the staff of the Huddersfield Royal Infirmary. The remaining thirty were sent to Durker Roods, Meltham, near Huddersfield. The men were met at the station (the near platform of which was cleared except of officials) by the Mayor, Mr. J. E. Willans, one of the governors of the infirmary, Mr. J. Bate, secretary, Dr. E. G. Coward (chief officer of the Voluntary Aid Detachment of the St. John Ambulance Association), Dr. E. W. N. Wooler, house surgeon at the infirmary, Colonel W. Cooper, Captain Wrigley, and Lieut. Freeman. There were twenty-eight members of the Voluntary Aid Detachment of the St. John Ambulance Association present under Dr. Coward and Superintendent G. W. Haigh. The arrangements for the men's accommodation at the Royal Infirmary were complete about three weeks ago.

### NORWICH: AN URGENT SUMMONS.

A third convoy of 100 wounded soldiers arrived at the above hospital at five o'clock last Sunday morning, the institution having been warned by wire on the previous Thursday as follows: "Beds urgently needed. Make as many as possible for oversea patients by every means in your power."

Of some 600 wounded collected from the battlefields of Northern France, brought from Boulogne to Southampton, the first contingent to be sent away were 100 cases allotted to the County Hospital at Norwich, and in seven and a-half hours the train arrived from Southampton without break or change. The proportion of serious injuries was greater than in any of the previous contingents, there being twenty-five cot cases. By 6.30 a.m. the whole party had been dispatched to the hospital, and a new chapter had been opened in the era of tremendous stress and responsibility imposed by this ghastly war upon the hospital.

For the first time the hospital has unfortunately to record the loss of two of the wounded. Ten minutes after the arrival of the men on Sunday morning Private J. Cannon, of the Royal Lancaster Regiment, whose home address is at Liverpool, called the Secretary to his bedside and calmly and collectedly told the address of his wife in order that she might be summoned to him by telegram. Ten minutes later he had passed away in the presence of the doctors, who speak of him in terms of the highest admiration. It was the deathbed of a hero facing the last enemy with the colours flying. The second patient, Private Edridge, of the 2nd Border Regiment, succumbed to an abdominal wound, which from the first had given the surgeons cause for grave misgiving.

There is at least one other case which should be recorded. A patient was brought in with a hole at the base of his right ear. A bullet had entered and become lost in his skull. Pending an examination under the *x*-rays he went to the bathroom after he arrived, and there, in the presence of witnesses, he had an experience odder than anything achieved at the recent hospital performance by Maskelyne and Devant. Feeling a little snuffly, as he describes it, at the top of his nose, he made an appropriate gurgitation, and the bullet came down his left nostril. There are two kinds of trophy in which these war-patients are particularly interested—metal taken out of their bodies and radiographs showing where metal still remains and the nature of the injuries thence arising.

There are now 147 wounded soldiers in this hospital; there are, however, many others at various convalescent homes and recognised Red Cross hospitals in the county. This hospital has been particularly fortunate in receiving offers from all parts of the county to take convalescent soldiers. Some mention ought to be made of the valuable services rendered by the generous people who are making themselves in many instances wholly responsible for the entire up-keep of convalescent homes for wounded soldiers from the Front. Wounded soldiers are being sent direct to some convalescent homes in the county by the War Office authorities.

### NORTH STAFFORDSHIRE INFIRMARY, STOKE-ON-TRENT.

In the early hours of Sunday, November 1, an ambulance train, which had left Southampton on the previous night and detrained a portion of its load at Birmingham *en route*, arrived at Stoke-on-Trent with seventy-seven wounded British soldiers direct from the fighting line in Belgium. Forty-seven of this number were "stretcher" cases, but in a short space of time the whole convoy of wounded had been conveyed to the hospital by means of motor-cars, under the superintendence of Dr. H. H. Folker, Assistant Commissioner for Red Cross in Staffordshire. Many had trophies to show of personal struggles and triumphs. The wonderful efficiency of the British transport arrangements is seen in the fact that these men, most of them wounded on or about Thursday evening last week in the neighbourhood of Ypres, should be safely lying in hospital in the north of Staffordshire by 8 a.m. on the following Sunday.

**[A few accounts are unavoidably held over.]**

